# From the anal canal to the pancreas: diagnostic challenges of radiologic surveillance in detecting rare but potentially curable metastases — a case report

**DOI:** 10.3389/fonc.2025.1651773

**Published:** 2026-01-21

**Authors:** Jelena Stanić, Ivana Šović, Luka Jovanović, Predrag Nikić, Tatjana Arsenijević

**Affiliations:** 1 Faculty of Medicine, University of Belgrade, Belgrade, Serbia; 2 Department of Radiation Oncology, Institute for Oncology and Radiology of Serbia, Belgrade, Serbia; 3 Department of Radiological Diagnostics, Institute for Oncology and Radiology of Serbia, Belgrade, Serbia; 4 Clinic of Urology, University Clinical Center of Serbia, Belgrade, Serbia

**Keywords:** anal squamous cell carcinoma, chemoradiotherapy, diagnostic accuracy, oncologic surveillance, pancreatic metastasis, rare metastasis

## Abstract

**Introduction:**

Pancreatic metastases from anal canal squamous cell carcinoma (SCC) are exceptionally rare. To date, only one previous case has been reported. The present case represents the second documented instance, highlighting the unusual metastatic pattern and the challenges in diagnosis and treatment. Due to the rarity of such metastases, their clinical presentation and optimal management remain poorly understood.

**Case presentation:**

We describe a 64-year-old woman diagnosed with locally advanced anal canal SCC, with diagnosis delayed as she had deferred medical consultation, who then received definitive chemoradiotherapy. During routine follow-up imaging, a small pancreatic lesion was initially overlooked and not reported. One and a half years later, the patient presented with nonspecific abdominal symptoms, and imaging revealed a 5 cm pancreatic mass. Endoscopic ultrasound-guided biopsy confirmed metastatic SCC of anal origin. The mass was considered inoperable due to vascular invasion. Systemic chemotherapy induced partial remission; however, due to hematologic toxicity, treatment was modified, and stereotactic body radiotherapy was applied to the pancreatic lesion, resulting in disease stabilization. Subsequent pulmonary metastases were treated with second-line chemotherapy, resulting in further partial remission; however, later disease progression was observed, limited to the pancreas, and third-line chemotherapy was planned but not initiated due to deterioration of general condition.

**Conclusion:**

This case highlights the importance of vigilant long-term surveillance and multidisciplinary management in patients with anal SCC, especially when atypical metastatic sites like the pancreas are involved. Early identification and histopathological confirmation enable timely, targeted treatment, which can improve clinical outcomes in such rare presentations.

## Highlights

- Pancreatic metastases from anal canal SCC are extremely rare and often present atypically, necessitating vigilant and comprehensive diagnostic evaluation.
- Early detection through advanced imaging and histopathologic confirmation is critical for timely and effective management.
- Long-term, structured oncologic follow-up with appropriate imaging is essential to identify uncommon metastatic spread and improve patient outcomes.

## 1 Introduction

Anal cancer accounts for approximately 2% of all gastrointestinal malignancies and less than 7% of anorectal cancers. The median age at diagnosis is 60 years, and it is rarely seen in individuals under the age of 35. Squamous cell carcinoma (SCC) is the most common histological subtype of anal cancer (1).

Anal cancer typically spreads locally to adjacent structures, such as the rectum and perianal skin, and exhibits a relatively low rate of distant metastasis. When distant spread does occur, it most frequently involves the liver, lungs, and distant lymph nodes (2, 3). Pancreatic metastases from anal canal SCC are exceedingly rare. In general, metastases to the pancreas from other primary malignancies are themselves uncommon, representing only about 2% of all pancreatic malignancies (4). Although isolated pancreatic metastases have been reported, they are typically incidental findings in patients with widespread disease (5–7). The kidney, breast, colon, skin, and lung are the most common primary sites of malignancy that metastasize to the pancreas (2, 5). While rare, pancreatic metastases from rectal cancer have also been documented (8–12). To the best of current knowledge, only one previous case of pancreatic metastasis from anal canal SCC has been reported (Duzdar & Gromski, 2021). In that case, a 66-year-old woman presented with dizziness and syncope, and imaging revealed a solitary pancreatic mass with portal vein involvement. She had a history of a positive fecal immunochemical test four months earlier but refused a colonoscopy at that time. Endoscopic ultrasound-guided fine needle biopsy (EUS-FNAB) later confirmed anal SCC, and flexible sigmoidoscopy demonstrated a 4 cm fungating anal canal mass. The patient ultimately received hospice care. The present case therefore represents the second documented instance of pancreatic metastasis from anal canal SCC (7).

Here, we present a rare case of pancreatic metastasis originating from anal canal SCC, along with a review of the relevant literature. Early recognition of such atypical metastatic presentations is critical, especially when the pancreas is the only site of distant disease and the patient remains in good general condition, as timely diagnosis may allow for potentially curative treatment.

## 2 Case presentation

We report the case of a 64-year-old woman who developed a perineal lump over several months, which she initially self-attributed to hemorrhoids, resulting in a delay in seeking medical evaluation. Her medical history included neoadjuvant chemotherapy followed by quadrantectomy for right-sided breast cancer, along with five years of adjuvant tamoxifen therapy initiated in 2000. There was no significant family history of hereditary cancers. Her most recent routine checkup with a general practitioner, performed in April 2016, had shown no abnormalities.

**Figure 1:**
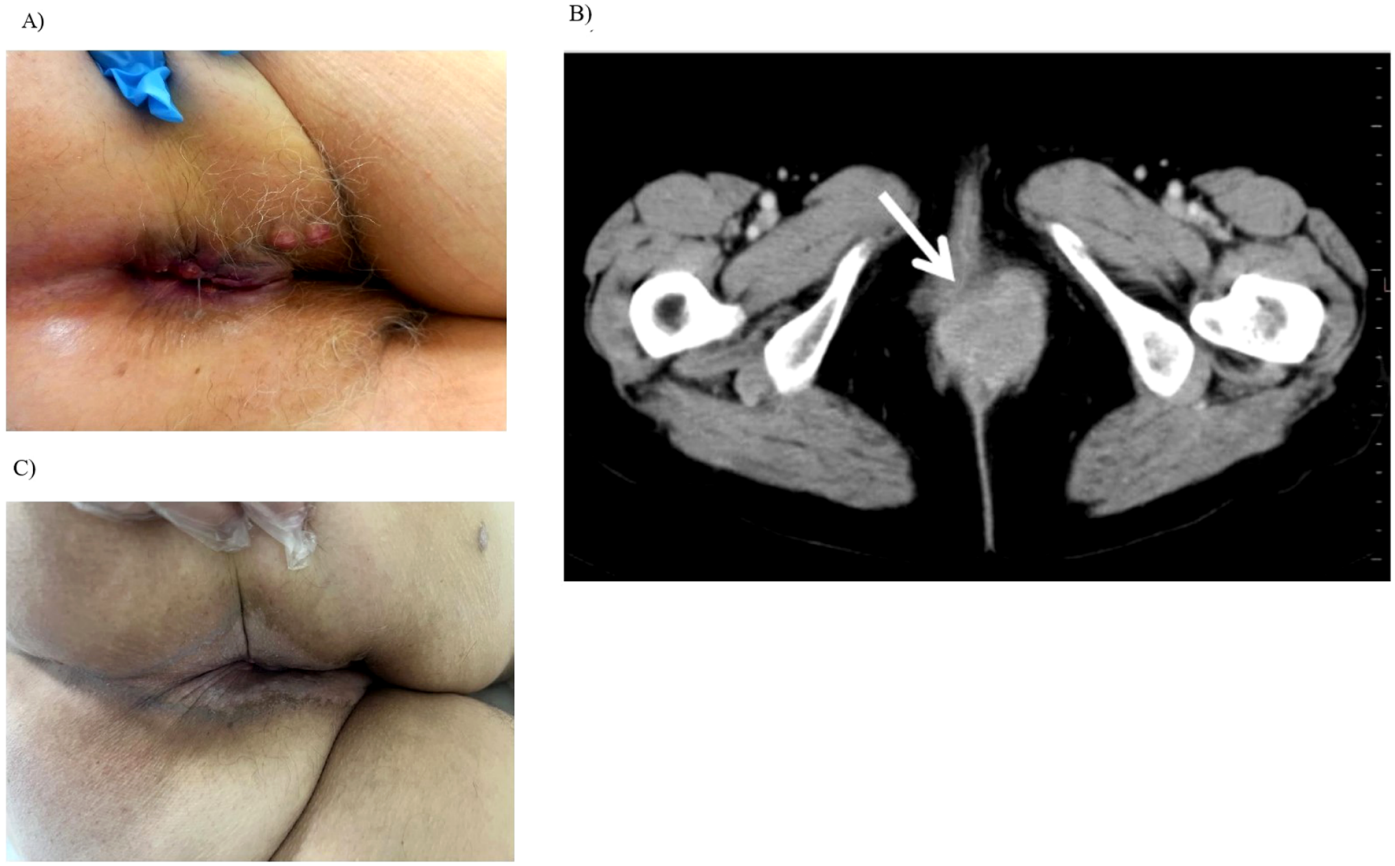
Clinical and radiological findings: Local effects before and after CRT **(A)** Clinical examination showing an extensive anal canal tumor with contiguous spread to the labia majora and adjacent perianal skin. **(B)** Axial CT scan demonstrating a bulky anal canal mass with proximal extension into the rectum and anterior infiltration of the labia majora. **(C)** Clinical presentation after CRT showing complete local response. The previously extensive lesion has fully resolved, with no visible or palpable signs of disease.

The patient presented to the Institute for Oncology and Radiology of Serbia in February 2020 with symptoms including a palpable mass at the anal verge, rectal bleeding, and anal pain. Notably, she had experienced marked unintentional weight loss, decreasing from 65 kg to 52 kg over a few months. A clinical examination revealed a large, invasive, exophytic tumor involving the perianal skin and extending to the vaginal introitus. Complete blood count (CBC) and laboratory values were within normal limits. Tumor markers, including CA 15-3, CA 19-9, and carcinoembryonic antigen (CEA), were also normal. Digital rectal examination (DRE) detected a palpable tumor in the anal canal, extending into the rectum and bleeding upon contact (**Figure 1A**).

Endoscopic examination confirmed the presence of an anorectal mass. Histopathological analysis performed at the Institute of Pathology, Faculty of Medicine, University of Belgrade, revealed squamous cell carcinoma (SCC), Grade 2. Computed tomography (CT) of the chest, abdomen, and pelvis demonstrated tumor extension from the perineum into the rectum, with infiltration of the vaginal introitus and vulva. Enlarged mesorectal and inguinal lymph nodes were identified, with no evidence of distant metastases. Accordingly, the disease was staged as cT4N1aM0 (**Figure 1B**). Although pelvic magnetic resonance imaging (MRI) is recommended by NCCN guidelines (13) for local staging, it could not be performed at the time of diagnosis due to technical constraints and prolonged waiting lists related to the COVID-19 pandemic. Given that MRI findings were not expected to modify the treatment strategy or impact clinical decision-making in the context of already locally advanced disease, pelvic MRI was therefore deferred.

In May 2020, the multidisciplinary tumor board at the Institute for Oncology and Radiology of Serbia recommended definitive chemoradiotherapy (CRT), based on the patient’s excellent general condition and Eastern Cooperative Oncology Group (ECOG) performance status of 0. Informed consent was obtained. In August 2020, she began primary radiotherapy (RT) combined with concurrent chemotherapy (CHT), consisting of 5-fluorouracil (5-FU) and cisplatin, in accordance with the established treatment protocol followed at our institution. Radiotherapy was delivered using volumetric modulated arc therapy (VMAT), with a total dose of 59. 4 Gy in 33 fractions to the primary tumor using conventional fractionation (1. 8 Gy per fraction). Involved lymph nodes received 50. 4 Gy. Concurrent chemotherapy included 5-FU administered via continuous infusion on days 1–4 and 29–32 (1000 mg/m²/day), and cisplatin given on days 1 and 29 (100 mg/m²).

During the first CHT cycle, the patient developed grade 4 neutropenia, grade 2 anemia, and grade 2 radiation dermatitis, per Common Toxicity Criteria (CTC) (14). She received supportive care, including granulocyte colony-stimulating factor (G-CSF), broad-spectrum antibiotics, and red blood cell transfusions. Due to significant hematologic toxicity, the second CHT cycle was omitted, extending the total treatment duration. Definitive CRT was completed over 81 days, from August 25 to November 13, 2020.

At the initial follow-up in March 2021, chest CT and abdominal-pelvic MRI were performed. Although abdominal MRI is not routinely recommended for follow-up after CRT for anal canal cancer according to NCCN guidelines (13), it was conducted in this case due to the initially T4 stage of the tumor, the MRI being available as it had been scheduled immediately after the completion of CRT, and the patient’s prior history of breast cancer, which had not been regularly monitored after treatment. Both examinations demonstrated a complete response, confirming the effectiveness of the therapy. The patient reported no fecal incontinence or external anal sphincter dysfunction, and physical examination and anoscopy revealed no evidence of local recurrence (**Figure 1C**).

At the October 2021 follow-up, only pelvic MRI was performed, as the previous abdominal-pelvic MRI had shown no signs of disease. This examination again revealed no evidence of recurrence, confirming the ongoing complete response.

In May 2022, she presented to the emergency department with a two- to three-week history of nausea, anorexia, and diffuse abdominal pain radiating to the left scapula and mid-back. On physical examination, vital signs were stable, and laboratory tests showed elevated inflammatory markers, lipase, and amylase levels; the patient was treated under the diagnosis of acute pancreatitis. No abdominal CT or MRI was performed at that time. The patient responded to conservative management, which led to her discharge without further diagnostic evaluation. The patient did not adhere to oncologic follow-up after the episode of acute pancreatitis and did not seek oncologic evaluation until September 2022.

**Figure 2:**
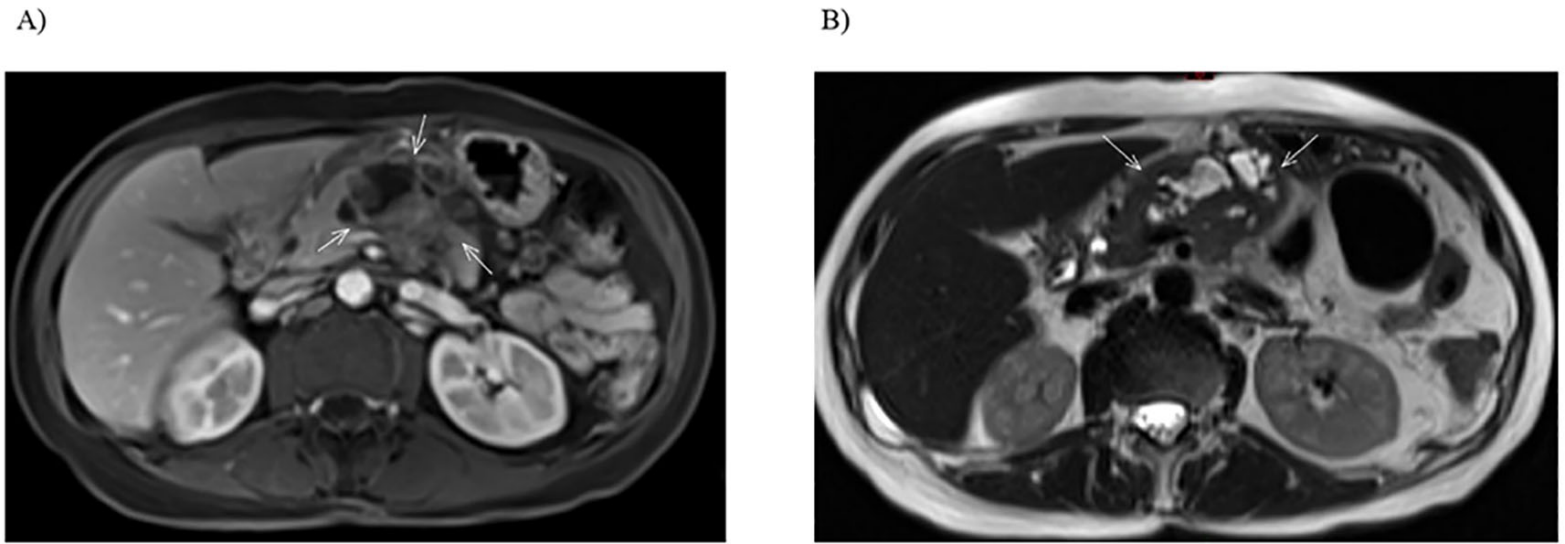
Imaging of the pancreatic lesion before biopsy. **(A)** Axial T1-weighted post-contrast MRI shows a solid-cystic tumor in the pancreatic body, with invasion of the superior mesenteric vein and anterior extension towards the abdominal wall. **(B)** Axial T2-weighted MRI highlights the cystic component of the lesion and demonstrates infiltration into the jejunal loop.

In September 2022, following recurrent episodes of pancreatitis-like symptoms and in the context of her prior cancer history, the patient herself consulted her oncologist. She was referred for an abdominal-pelvic MRI, which revealed a 5 cm mass in the pancreatic body, extending to the anterior abdominal wall and invading the superior mesenteric vein and adjacent jejunal loops. At the same time, the previously treated anal canal remained in complete local response, with no evidence of residual or recurrent tumor, concomitant chest CT was unremarkable. The findings were considered highly suspicious for either a primary pancreatic malignancy or metastatic involvement. Despite the concerning imaging features, tumor markers remained within normal limits (**Figures 2A, B**).

**Figure 3:**
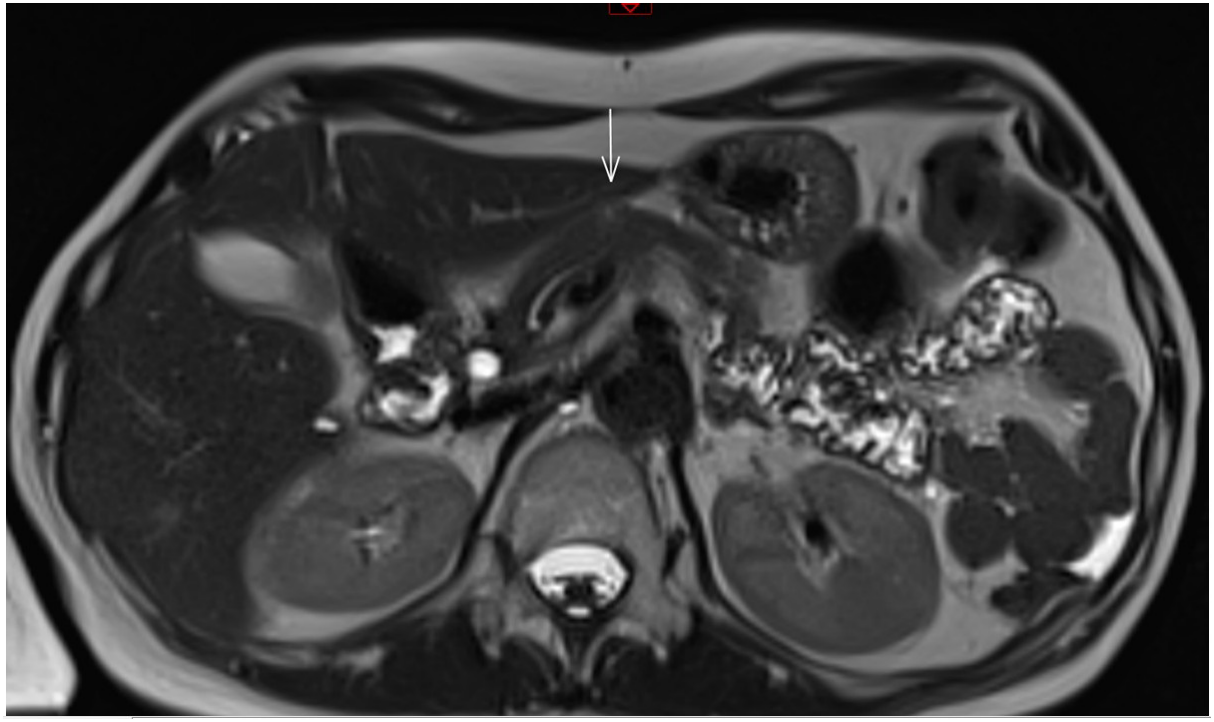
Initial presentation of pancreatic metastasis. Focal subcentimeter lesion on the ventral aspect of the pancreatic body, showing no diffusion restriction or post-contrast enhancement on axial T1-weighted images, and appearing isointense on axial T2-weighted images.

In light of the MRI findings in September 2022, a retrospective review of all prior diagnostic imaging was performed. This review revealed that at the initial follow-up in March 2021, abdominal MRI had shown a focal subcentimeter lesion on the ventral aspect of the pancreatic body, which did not demonstrate diffusion restriction nor post-contrast enhancement on T1-weighted images, and appearing isointense on axial T2-weighted images. Likely due to the complete response at that time, the small lesion was overlooked by the radiologists and was therefore not followed. At that time, even if the lesion had been recognized and noted, it could not be determined whether it was benign or malignant (**Figure 3**).

Surgical evaluation in October 2022 deemed the pancreatic mass inoperable. The patient had no other signs of disease and was in excellent general condition. Given her history of both breast and anal SCC, and considering that the radiologist had described the lesion as highly suspicious for either a primary tumor or metastasis, histopathological evaluation was advised. At the Clinic for Digestive Surgery, First Surgical Clinic, University Clinical Center of Serbia, Belgrade, EUS-FNAB was performed. Pathological analysis at the Institute of Pathology, Faculty of Medicine, University of Belgrade, confirmed that the lesion was a metastasis from anal canal SCC.

**Figure 4:**
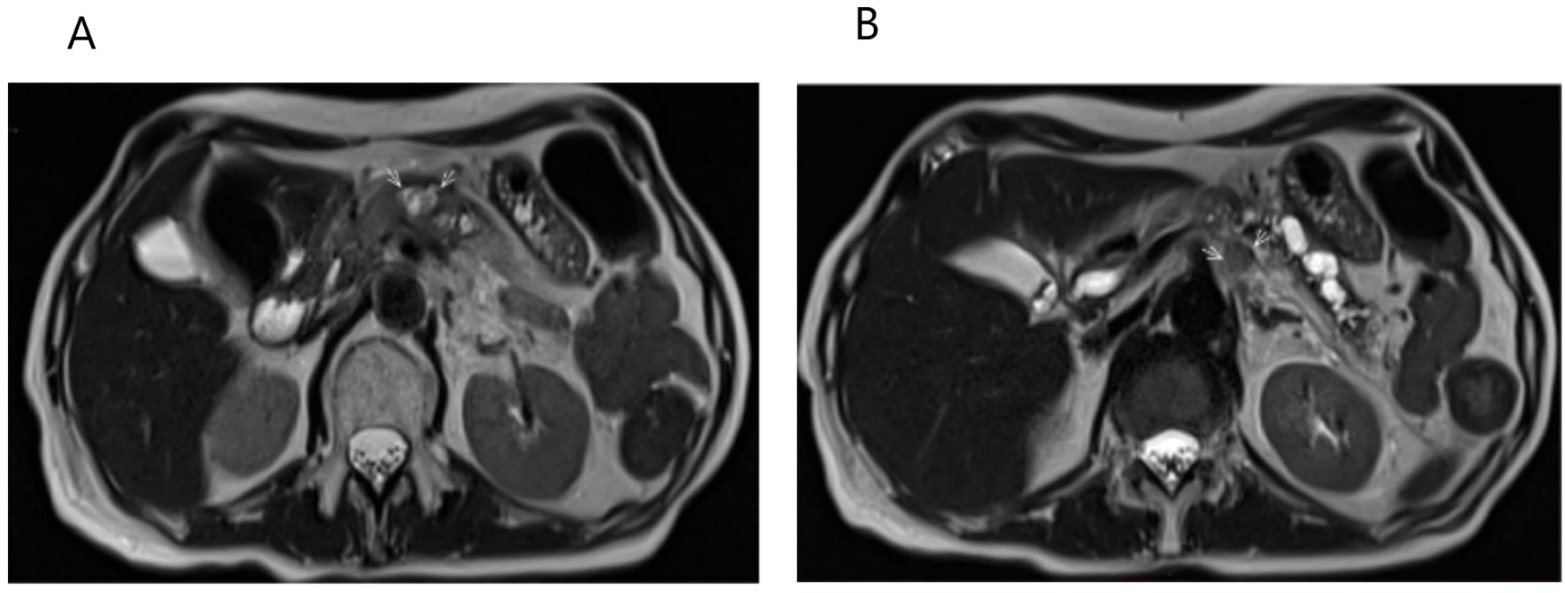
Radiologic evaluation of the pancreatic lesion and regional lymph node after systemic chemotherapy. **(A)** Axial T2-weighted MRI reveals an oval, heterogeneous area measuring 1.8 x 2.4 cm, containing small necrotic foci and restricted water diffusion, indicating residual active tumor tissue. **(B)** Axial T2-weighted MRI shows a slightly enlarged oval lymph node near the celiac trunk, measuring 1.3 x 1.1 cm, also with restricted water diffusion.

In November 2022, the patient, who was ECOG 1 with preserved organ function, was evaluated by the multidisciplinary tumor board, which recommended systemic CHT with mitomycin and a fluoropyrimidine. She completed nine cycles of CHT, concluding in July 2023. Hematologic toxicities included grade 1 leukopenia, grade 3 anemia, and grade 1 thrombocytopenia, which prompted discontinuation of further treatment. Follow-up abdominal-pelvic MRI demonstrated substantial tumor regression, consistent with partial remission, and chest CT showed no signs of the disease (**Figures 4A, B**).

**Figure 5:**
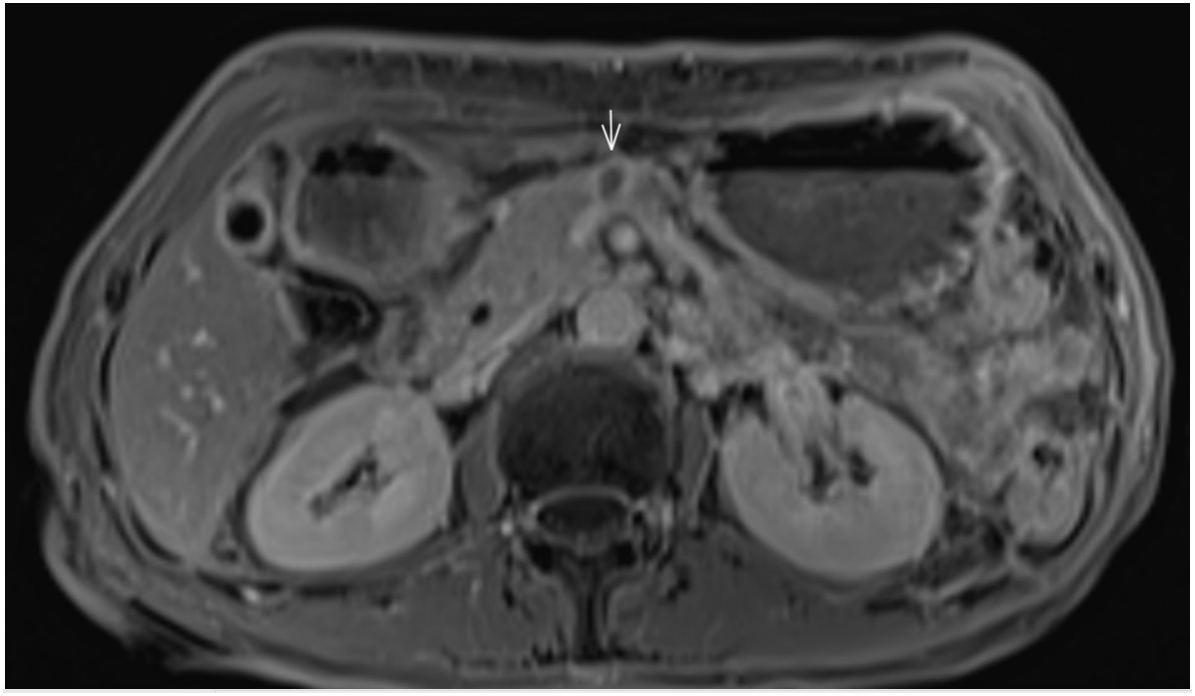
Stabilization of pancreatic mass following SBRT. Axial post-contrast T1-weighted MRI demonstrates a 1 x 1,6 cm focal lesion in the pancreatic body following SBRT, with central necrosis and peripheral signal intensity enhancement, assessed as stable disease.

Given that the pancreatic lesion was the only site of disease, stereotactic body radiotherapy (SBRT) was administered to the lesion in January 2024, delivering a total dose of 30 Gy in 5 fractions. A July 2024 follow-up abdominal-pelvic MRI demonstrated stable disease in the pancreas with no evidence of pelvic recurrence (**Figure 5**).

In September 2024, evaluation was prompted by intermittent fatigue and dyspnea. Chest CT and abdominal MRI revealed pulmonary metastases and enlargement of the pancreatic lesion, now measuring 3. 5 × 2. 8 cm with diffusion restriction. Due to prior treatment-related toxicity, the tumor board recommended second-line CHT with paclitaxel and carboplatin. Treatment began in October 2024.

After six cycles of chemotherapy, a follow-up chest, abdominal and pelvic CT in February 2025, performed as the only available imaging modality, as MRI was not consistently available, demonstrated a partial response in both the lungs and the pancreas. Chemotherapy was subsequently continued as monotherapy with paclitaxel until April 2025, after which the patient was transitioned to a regimen of regular follow-up.

**Figure 6:**
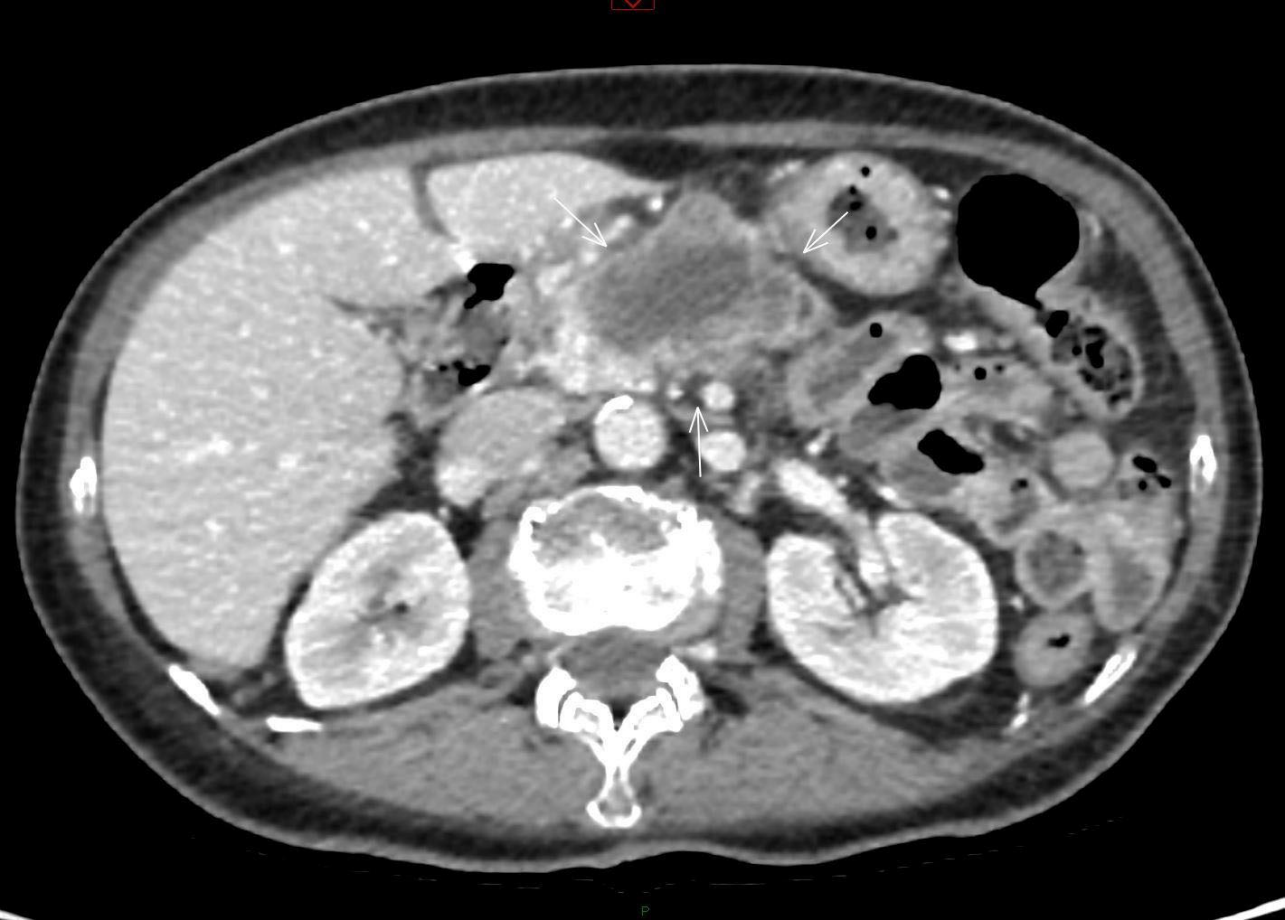
Radiologic progression of pancreatic metastasis after second-line chemotherapy. Contrast-enhanced CT shows a hypodense, irregular, heterogeneous mass in the pancreatic body junction, measuring 5.2 × 4.4 cm, with distal pancreatic atrophy and main pancreatic duct dilatation. The lesion invades the splenic and superior mesenteric veins, demonstrates approximately 180° circumferential contact with the splenic artery, and abuts the left hepatic lobe.

At the first follow-up in July 2025, progression was observed on CT. In the region of the pancreatic head and body, there was a hypodense, irregular, heterogeneous lesion measuring 5. 2 × 4. 4 cm (**Figure 6**). The patient was referred to a multidisciplinary tumor board on July 23, 2025, and the decision was made to initiate retreatment with monotherapy mitomycin.

The patient’s perspective is clearly stated, including her commitment to continue treatment and her tolerance of therapy. However, due to deterioration in her overall health, with notable weakness and malaise, the planned therapy has not yet been initiated. Symptomatic and supportive care is ongoing. **Table 1** summarizes the case timeline.

**Table 1:** Case timeline.

Date	Clinical/Symptoms	Imaging/diagnostic evaluation	Histopathology/Cytology	Therapy/key interventions
February 2020	Perianal mass, rectal bleeding, anal pain, significant weight loss	Chest, Abdomen, Pelvis CT	Biopsy: Grade 2 anal SCC	MDTB decision for definitive CRT
August 2020 - November 2020	–	–	–	Definitive CRT (1st cycle of 5-FU + cisplatin with VMAT RT); treatment complicated by hematologic toxicity
March 2021	Asymptomatic	Chest CT and abdominal–pelvic MRI: CR; subcentimeter pancreatic lesion overlooked and noted retrospectively	–	Follow-up and observation
October 2021	Asymptomatic	Pelvic MRI: CR	–	Follow-up and observation
May 2022	Nausea, anorexia, diffuse abdominal pain	–	–	Suspected pancreatitis; conservative management with partial symptom relief
September-October 2022	Recurrent pancreatitis-like symptoms	Abdominal-pelvic MRI: 5 cm pancreatic mass, likely progression of previously overlooked subcentimeter lesion; CR at anal canal; Chest CT: no dissemination	–	Surgical evaluation; lesion deemed inoperable
October 2022	–	EUS-FNAB	Metastatic SCC of anal canal origin	–
November 2022	–	–	–	MDTB decision for first line of systemic CHT
November 2022 - July 2023	–	Follow-up abdominal-pelvic MRI: PR of pancreatic lesion chest CT: no dissemination	–	9 cycles of first line systemic CHT (mitomycin + fluoropyrimidine)
January 2024	–	–	–	SBRT to pancreatic lesion (30 Gy in 5 fractions)
July 2024	–	Abdominal-pelvic MRI: SD of pancreatic metastasis	–	Continued follow-up
September 2024-February 2025	Fatigue, dyspnea	Chest CT and abdominal MRI: pulmonary metastases and PD of pancreatic metastasis	–	MDTB decision for second line systemic CHT (6 cycles of paclitaxel + carboplatin)
February-April 2025	–	Chest, Abdomen, Pelvis CT: PR in lungs and pancreas	–	Continued mono-CHT (3 cycles of paclitaxel)
July 2025	–	Chest, Abdomen, Pelvis CT: PD of disease in pancreas, CR in lungs	–	MDTB decision for third line systemic CHT (monotherapy with mytomicin)
Avgust 2025	Generalized weakness, malaise, and cachexia	–	–	CHT not initiated (worsening general condition); palliative/supportive therapy ongoing

CRT , Chemoradiotherapy; CHT, Chemotherapy; RT, Radiotherapy; VMAT, Volumetric Modulated Arc Therapy; SBRT, Stereotactic Body Radiotherapy; MRI, Magnetic Resonance Imaging; CT, Computed Tomography; EUS-FNAB, Endoscopic Ultrasound-Guided Fine Needle Aspiration Biopsy; CR, Complete Response; PR, Partial Response; SD, Stable Disease; PD, Progressive Disease; SCC , Squamous Cell Carcinoma; MDTB, Multidisciplinary Tumor Board.

## 3 Discussion

We have presented a rare case of pancreatic metastasis originating from anal cancer, an exceedingly uncommon event in clinical oncology. This case underscores the need to consider atypical metastatic pathways, even in cancers that are themselves relatively rare, such as anal SCC. The development of a pancreatic metastasis in our patient highlights the complexity of metastatic progression and the importance of a thorough diagnostic workup. When a solitary pancreatic mass is detected, the differential diagnosis should include the possibility of metastatic disease, particularly anal SCC, especially if the clinical presentation is suggestive, as in this case (5).

Despite its classification as a rare malignancy, the incidence of anal cancer in the United States has steadily increased over the past several decades. Recent data indicate that the incidence of anal SCC is rising by approximately 2. 7–2. 9% annually, particularly among individuals aged 50 years and older. Mortality rates have shown a parallel increase, with an average annual rise of 3. 1% (13). Clinical recognition and diagnostic confirmation remain challenging due to the tumor’s rarity and nonspecific presentation.

Perianal masses can mimic a broad spectrum of benign and malignant conditions. Thrombosed hemorrhoids typically present as acutely painful, firm nodules, whereas perianal abscesses or fistulas manifest with pain, swelling, or drainage. Primary cutaneous malignancies may appear as progressively enlarging, ulcerated, or bleeding lesions, while rare soft tissue sarcomas often present as firm, painless masses with subtle skin changes. Inflammatory and infectious conditions, including perianal dermatitis, hidradenitis suppurativa, Crohn’s disease-related perianal involvement, and bacterial, viral, or fungal infections such as condyloma acuminatum, as well as pilonidal cysts or abscesses, should also be considered, as they may closely resemble both benign and malignant entities (15). A systematic diagnostic approach, integrating careful visual inspection, DRE, and imaging when indicated, is essential for narrowing the differential diagnosis. Early histopathological assessment through biopsy of atypical or suspicious perianal lesions is critical, as timely evaluation enables accurate diagnosis and guides appropriate oncologic management (15, 16). This case highlights the consequences of delayed presentation, as our patient sought medical attention only after the lesion had enlarged and become associated with rectal bleeding and anal pain. It underscores the importance of early consultation, meticulous perianal examination, and prompt biopsy of atypical or progressive lesions to establish an early and accurate diagnosis and facilitate optimal oncologic management (17).

Pancreatic metastasis remains an unusual occurrence, representing approximately 2% of all pancreatic malignancies, most commonly originating from renal cell carcinoma, lung, breast, colorectal cancer, or melanoma. Anal canal SCC, which accounts for a small subset of gastrointestinal cancers, rarely metastasizes distantly and typically spreads to regional lymph nodes and the liver, followed by non-regional lymph nodes, bone, kidney, and peritoneum (2, 3, 13, 18, 19). The case we describe is exceptionally rare. To date, only one previous case has been documented in the literature, making this the second reported instance of such a metastasis (7). While both cases involved solitary pancreatic metastases from anal SCC, differences in patient symptoms and treatment decisions provide valuable insight. Reporting such rare cases raises clinician awareness and highlights the importance of timely diagnosis, as isolated pancreatic metastases from anal canal SCC may be curatively treated if detected early (2, 20–22).

In our patient, the initial presentation included recurrent pancreatitis-like symptoms and vague gastrointestinal complaints, which contributed to a delay in identifying metastatic disease. This mirrors findings in the literature, where pancreatic lesions are often incidentally discovered or misdiagnosed until confirmed by advanced imaging or histopathologic evaluation (6–12). Consistent with the observations by Karageorgou et al., tumor markers such as CA 19–9 and CEA are frequently within normal ranges in patients with pancreatic metastases from colorectal cancer, limiting their diagnostic value (11). Therefore, a combination of imaging and tissue confirmation is essential for accurate diagnosis.

Patients with isolated pancreatic metastases from colorectal origin may present with few or nonspecific symptoms, contrasting with primary pancreatic cancers, which are more often associated with abdominal pain, weight loss, and obstructive jaundice. In a series by Chai-Wei Lee et al., 45% of patients with pancreatic metastases were asymptomatic at presentation, underscoring the potential for silent progression (8). Moreover, the imaging features of primary and metastatic pancreatic lesions can overlap. Findings such as pancreatic ductal dilatation, obstructive jaundice, or pancreatitis may erroneously suggest a primary malignancy (8–10).

**Figure 7:**
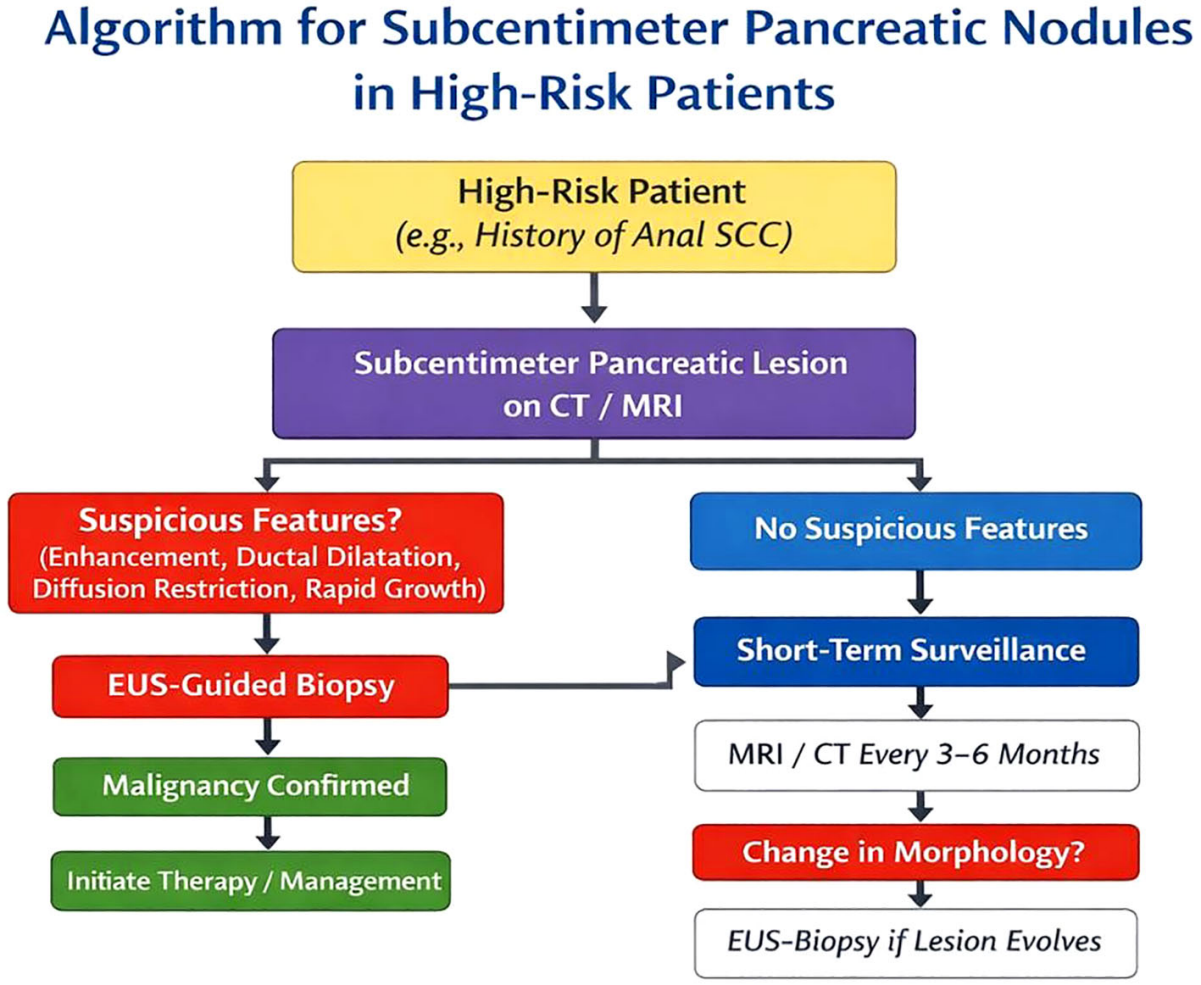
Algorithm for diagnosis and management of subcentimeter pancreatic lesions. Diagnostic and management algorithm for pancreatic cystic lesions, adapted from Schmitz et al. (24), with clinical management considerations informed by experience with isolated pancreatic metastases from colorectal malignancies (8).

The differential diagnosis of subcentimeter pancreatic lesions is particularly challenging in patients with a history of malignancy (23). Benign entities such as small pancreatic cysts (including low-risk intraductal papillary mucinous neoplasms and serous cystadenomas), post-pancreatitis pseudocysts, focal fatty infiltration, and intrapancreatic accessory spleen must be considered alongside malignant lesions, including small neuroendocrine tumors, early pancreatic adenocarcinoma, and metastatic disease (24, 25). Cross-sectional imaging with contrast-enhanced CT or MRI is essential for lesion detection; however, subcentimeter lesions frequently demonstrate nonspecific imaging characteristics and may lack ductal obstruction or a desmoplastic reaction, thereby limiting diagnostic specificity (26). According to NCCN guidelines, dedicated pancreatic-protocol CT or MRI is recommended for pancreatic lesion characterization and staging. Endoscopic ultrasound (EUS)–guided biopsy should be considered when imaging findings are indeterminate or when histological confirmation may influence clinical management (27). The integration of vigilant imaging follow-up with timely histopathological assessment enables early detection of rare metastatic events and supports informed clinical decision-making, as illustrated in the present case (27–29). In patients with subcentimeter pancreatic lesions, close surveillance with short-interval imaging is recommended to monitor interval growth or morphological changes. This typically includes abdominal and pelvic MRI every three to six months, along with annual thoracic CT imaging (28, 30, 31) (**Figure 7**). Although distant metastatic spread is uncommon, atypical relapse patterns may warrant additional diagnostic evaluation. According to NCCN guidelines, ^18F-fluorodeoxyglucose positron emission tomography/computed tomography (FDG PET/CT) is not recommended for routine surveillance but may be considered in cases of suspected metastatic disease or equivocal findings on conventional imaging, particularly when recurrence occurs at unusual sites.

In the present case, endoscopic ultrasound (EUS)-guided biopsy was crucial in establishing the final diagnosis and guiding subsequent treatment decisions. Furthermore, comprehensive histological and immunohistochemical profiling was of particular importance, given the patient’s history of two distinct malignancies (13, 18, 19).

With regard to treatment, previous studies have shown that isolated pancreatic metastases may be surgically resectable in carefully selected patients, particularly those with renal cell carcinoma or colorectal cancer. Resectability is generally determined by the patient’s performance status, absence of major vascular invasion, and technical feasibility, rather than by a strict size cutoff (32–35). However, due to the extreme rarity of pancreatic metastasis from anal SCC, no standardized treatment approach exists. Most available evidence comes from small case series and retrospective reports, which limits the development of evidence-based therapeutic guidelines (17). In the published literature, most patients with pancreatic metastases from SCC, whether from the lung, cervix, esophagus, or anus, receive palliative treatment, although some case reports have suggested potential benefit from surgical intervention in highly selected patients with favorable performance status and no other metastatic disease (2, 21, 36).

In our case, surgery was not an option due to vascular invasion and local extension. Nonetheless, the patient’s preserved performance status (ECOG 1) and absence of comorbidities supported the decision to initiate systemic CHT. The initial response to mitomycin-C and fluoropyrimidine-based CHT was encouraging, with a partial radiographic response achieved. Although hematologic toxicity prevented continuation, this case supports findings that systemic therapy can provide disease control, even in rare metastatic patterns (13, 17).

Interestingly, the pancreatic metastasis remained radiologically stable for more than one year before pulmonary progression became evident (**Table 1**). This relatively indolent course raises the question of whether earlier or more aggressive local treatment, including surgical resection if technically feasible, might have achieved improved local control or conferred a survival benefit. A growing body of literature supports curative-intent resection in carefully selected patients with isolated pancreatic metastases, with the rationale of reducing tumor burden, delaying systemic progression, and potentially improving quality of life (2, 11).

Given our patient’s excellent initial response to definitive CRT and a prolonged disease-free interval, it is reasonable to speculate that curative resection of the pancreatic lesion could have favorably influenced the subsequent disease course had it been feasible in this case (21). While standard surveillance protocols primarily focus on detecting local and regional recurrence, uncommon patterns of distant metastasis can arise and should be considered, especially in patients presenting with atypical symptoms (8, 10, 21). Also, disease monitoring in this patient was complicated by technical limitations, including periods of limited access to MRI when clinically indicated, which represents a recognized limitation in the assessment of disease progression. In many healthcare systems, restricted availability of advanced diagnostic imaging continues to pose a challenge to optimal oncologic surveillance and timely clinical decision-making.

In recent years, several biomarkers have emerged as important prognostic factors in SCC, including HPV status, p16 expression, TP53 status, and PD-L1 expression. These biomarkers have demonstrated prognostic relevance and may help predict response to conventional treatments, with HPV-positive and p16-positive tumors generally exhibiting a more indolent clinical course and improved outcomes compared with tumors harboring TP53 alterations (37). Moreover, PD-L1 expression has been associated with improved local relapse-free survival and has been shown to correlate with HPV and p16 positivity, further highlighting the potential relevance of immunotherapeutic strategies in this patient population (37). Nevertheless, in our case molecular profiling won’t be of particular help since personalized therapeutic approaches are not routinely endorsed. Moreover, initial treatment with radical CRT will be largely the same regardless of HPV, TP53 and PD-L1 status.

The role of the multidisciplinary team (MDT) was central in managing this complex case. The patient’s care involved oncologists, radiologists, surgeons, pathologists, and supportive care providers. Coordinated MDT decision-making ensured accurate diagnostic workup, timely treatment planning, and flexibility in response to disease evolution. MDT discussions are particularly valuable in cases with atypical disease patterns, allowing for individualized, evidence-informed management (38).

Further research, including larger case series, is needed to better define the incidence, clinical behavior, and optimal management of pancreatic metastases from anal cancer. Future studies should aim to optimize imaging protocols for early detection. High-resolution imaging modalities such as CT and MRI, along with tissue sampling via EUS-FNAB combined with histopathological and immunohistochemical analysis, remain essential for accurate diagnosis, confirmation of metastatic origin, and guidance of treatment (32).

## 4 Conclusion

This case underscores the critical importance of meticulous diagnostic surveillance in patients with complex oncologic histories, particularly those with synchronous or metachronous malignancies. The progression of a previously incidental subcentimeter pancreatic lesion into a large, biopsy-confirmed metastatic deposit originating from anal canal SCC highlights the need for a high index of suspicion, even for initially nonspecific findings.

Early identification of isolated metastatic disease, especially when technically resectable, can create opportunities for curative treatment and significantly improve patient outcomes, as demonstrated in select cases from the literature. Nevertheless, this case also emphasizes the effectiveness of systemic therapy and the value of a MDT approach. Despite initial inoperability, the patient achieved a meaningful radiological response and maintained a favorable performance status for a sustained period through coordinated care and individualized treatment planning. Disease progression was observed later in the course of her illness.

## 5 Patient perspective

Despite limitations that precluded timely and comprehensive diagnostic evaluation, the patient survived for five years following the diagnosis of a second malignancy while maintaining a satisfactory quality of life. This was achieved through continued clinical follow-up and multidisciplinary management, which enabled symptom control, appropriate supportive interventions, and preservation of daily functioning and dignity throughout the disease course.

## Data Availability

The original contributions presented in the study are included in the article/supplementary material. Further inquiries can be directed to the corresponding author.
